# Comparative analysis on characteristics of two activated partial thromboplastin time reagents

**DOI:** 10.1002/jcla.24608

**Published:** 2022-07-19

**Authors:** Yutaro Yasui, Toshiaki Ishii, Junko Tatebe, Toshisuke Morita

**Affiliations:** ^1^ Department of Laboratory Medicine Toho University Graduate School of Medicine Tokyo Japan; ^2^ Department of Clinical Laboratory Toho University Omori Medical Center Tokyo Japan; ^3^ Department of Laboratory Medicine, Faculty of Medicine Toho University Tokyo Japan

**Keywords:** activated partial thromboplastin time, Coagpia APTT‐n, C‐reactive protein, multiple regression analysis, phospholipids

## Abstract

**Background:**

For the lack of standardized activated partial thromboplastin time (APTT), it has been pointed out that there are differences in values among several reagents. Recently, we have performed a parallel measurement on two reagents, Thrombocheck APTT‐SLA and Coagpia APTT‐n, and resulted with some dissociated samples. The purpose of this study is to clarify the possible factors related to ΔAPTT, the difference in measured values between the two reagents.

**Materials and Methods:**

In order to clarify the factors related to ΔAPTT, multiple regression analysis was performed on 8324 samples, using clinical laboratory data of all test items requested simultaneously with APTT. To confirm the items extracted from the multiple regression analysis, the target substance was spiked to pooled plasma and measured with two APTT reagents. Additionally, by spiking phospholipids, the effect on APTT measurement system was assessed.

**Result:**

Multiple regression analysis detected albumin–globulin ratio (AGR), C‐reactive protein (CRP), hematocrit, and prothrombin time as factors related to ΔAPTT (*p* < 0.001). Results revealed no significant differences when albumin was added to change the AGR. Whereas with the addition of CRP, prolongation of APTT was observed in Coagpia APTT‐n compared to Thrombocheck APTT‐SLA (*p* < 0.001). This prolongation was canceled by the addition of phospholipids, suggesting the interaction of CRP with phospholipids leads to the pseudo‐prolongation.

**Conclusion:**

It is considered that the pseudo‐prolongation of APTT is triggered by the interaction of CRP on the phospholipid in Coagpia APTT‐n, which contributed to the APTT dissociation.

## Graphical abstract

In multiple regression analysis, the difference of APTT value between the two APTT reagents showed a positive correlation with CRP. Additionally, an experiment of CRP addition revealed a significant prolongation of the measured values in APTT‐n in a CRP concentration‐dependent manner, and this prolongation by CRP was canceled by adding phospholipid, suggesting that CRP may inhibits phospholipid in APTT‐n reagent.

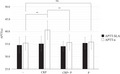

## 1 INTRODUCTION

Activated partial thromboplastin time (APTT) is the most commonly measured blood coagulation screening test along with prothrombin time (PT). It is used to evaluate the intrinsic and common pathway of coagulation. In addition to detect their abnormalities, it is used to extract lupus anticoagulant (LA) and inhibitors against coagulant factors.^1^, ^2^ Using a mixture of 3.2% sodium citrate and blood at a ratio of 1:9 as a sample, the principle of APTT starts with adding APTT reagent, containing contact activator and phospholipid, to the citrated plasma. After the factor activation during the incubation, calcium chloride is added which allows the formation of a clot. APTT is the time required until coagulation. However, according to the Clinical and Laboratory Standards Institute (CLSI), APTT should be detected as a prolonged coagulation time only when the activities of factor VIII, factor IX, and factor XI, are below 30% as numerical targets.^3^ It has been reported that the susceptibility to coagulation factors and drugs differ between APTT reagents, since there are a wide variety of activators and phospholipids which may be synthesized or originated from animals, causing the differences in the measured values.^4^, ^5^ This has been well documented in the past with regard to heparin^6^ and sensitivity to LA.^7^ However, APTT is used as heparin monitoring and an index of epidural anesthesia.^8^ Therefore, when a patient is transferred to a different hospital, it may be disadvantageous for patients to have different values depending on the measuring reagent.

Recently, for the purpose of changing APTT reagents, we have performed a parallel measurement on two reagents; Thrombocheck APTT‐SLA (hereinafter called APTT‐SLA) and Coagpia APTT‐n (hereinafter called APTT‐n), and resulted with some dissociated samples. Since these two reagents differ the phospholipids contained in the reagents as shown in Table 1, we predicted that the measured values diverge in certain pathological conditions with fluctuation of coagulation factor. Therefore, to clarify the factors causing the difference in the measured values of the two APTT reagents, we have conducted an exploratory study of the common characteristics of the dissociated samples, by utilizing the clinical laboratory data of all test items requested simultaneously with APTT.

**TABLE 1:** Activators and phospholipid sources in each APTT reagent

Reagent	Manufacturer	Phospholipid	Activator
Thrombocheck APTT‐SLA	Sysmex	Synthetic phosphatide	Ellagic acid
Coagpia APTT‐n	Sekisui Medical	Cephalin obtained from rabbit brain	Ellagic acid

## 2 MATERIALS AND METHODS

### 2.1 Subjects

Multiple regression analysis was performed on all clinical laboratory data of 8324 specimens requested concurrently with APTT, which were collected from the medical records of 3887 patients (1824 males and 2063 females) who underwent APTT testing at Toho University Medical Center in November and December 2016. Details about this study were disclosed in our institutional website and the potential participants were given the opportunity to decline to be further enrolled in the study (opt‐out). The study protocol was approved by the Ethics Committee of Toho University Omori Medical Center (No. M19214 17,298).

### 2.2 Measuring reagents and equipment

Two types of APTT reagents; Thrombocheck APTT‐SLA (Sysmex Corporation) and Coagpia APTT‐n (Sekisui Medical Co., Ltd) were used. Table 1 shows activators of the APTT reagents and the phospholipid composition. Fully automated coagulation analyzer CP3000 (Sekisui Medical Co., Ltd.) was used for the measurement of APTT. C‐reactive protein human recombinant (Oriental Yeast Co., Ltd.), human albumin (SIGMA‐ALDRICH), and hexagonal phospholipids (Staclot‐LA; Diagnostica Stago) were used in spiking experiments.

### 2.3 Sample preparation

Remnant specimens from inpatients and outpatients at our hospital, who have consented to comprehensive cooperation in the provision are centrifuged at 2000 × *g* for 10 min at 18°C and the plasma are collected as pooled samples and were used for the spiking experiments for the items extracted from the multiple regression analysis. CRP added samples were prepared by adding purified CRP to the pooled plasma, which the CRP value was within the reference range (CRP ≤ 1000 μg/L), and was adjusted to the amount of a change in CRP (ΔCRP) to 0, 12,500, 25,000, 50,000, and 100,000 μg/L. Albumin added samples were prepared by adding purified human ALB to the pooled plasma, which was prepared with ALB concentration of 20–30 g/L, and 5 s or more of a difference among the two APTT measured values, and was adjusted the volume of change in ALB (ΔALB) to 0 g/L, 5.0 g/dl, 10 g/L, and 20 g/L. Phospholipid added samples were prepared by mixing equivalent amount of pooled plasma and hexagonal phospholipids according to the attached document, and the purified CRP was added to adjust the ΔCRP to 0 μg/L, 0 mg/dl and 100,000 μg/L.

### 2.4 Statistical analysis

We examined the association of the APTT dissociation (APTT‐n measured value ‐ APTT‐SLA measured value; ΔAPTT) and the clinical laboratory data of other test items. However, since test items that are rarely performed cannot be used as explanatory variables for multiple regression analysis on account of the missing data; test items for which the number of requests less than 70% of that of APTT were excluded. First, we examined the relationship between the APTT dissociation and other test items, using univariate analysis. Spearman's rank correlation analysis was used, after confirming that there was no normality in the data distribution of ΔAPTT by performing Lilliefors test. Based on this analysis, in order to clarify the factors related to the difference in measured values between two APTT reagents, ΔAPTT was used as an objective variable, and a significant association was found with ΔAPTT in the univariate analysis, Moreover, multiple regression analysis was performed by the forced input method using variables with a correlation coefficient of | 0.2 | or more with ΔAPTT as explanatory variables, and factors affecting ΔAPTT were detected. In order to avoid multicollinearity, matrix correlation was performed between the explanatory variables, and only the items with the highest correlation with ΔAPTT among the items with a high correlation between the explanatory variables remained, and the rest were excluded. We also confirmed that the variance inflation factor: VIF of all input explanatory variables was less than 10. Data for test items are represented by the median (interquartile range). Bland–Altman analysis and Passing–Bablock regression were used to compare methods, and correlation was evaluated using Spearman's correlation coefficient. For fixed bias, we calculated the 95% confidence interval (CI) of the mean difference among measured values (95% limits of agreement). For proportional bias, we calculated Pearson's correlation coefficient. Paired *t* test was used to compare each phase of the prepared sample's APTT, APTT values, respectively. The above statistical analysis was performed using IBM SPSS Statistics version 25 and StatFlex version 6.0, and *p* < 0.05 was considered to be statistically significant.

## 3 RESULTS

### 3.1 Comparison of APTT‐n and APTT‐SLA measurements

**FIGURE 1:**
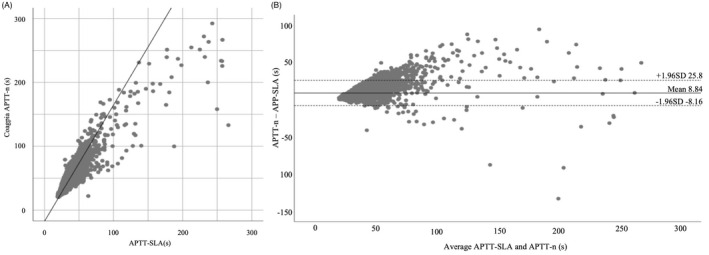
Correlation between APTT values of Thrombocheck APTT‐SLA and Coagpia APTT‐n (*n* = 8324). (A) Scatterplot showing the correlation between Thrombocheck APTT‐SLA (*x*‐axis) and Coagpia APTT‐n (*y*‐axis). The black line represents the Passing–Bablok regression line (equation: y = 1.82x−17.40, rS = 0.859, *p* < 0.001). (B) The Bland–Altman analysis. Dashed lines indicate upper and lower limits of agreement and solid line indicates the mean difference. APTT‐n, Coagpia APTT‐n; APTT‐SLA, Thrombocheck APTT‐SLA; rS, Spearman's rank correlation coefficient.

Passing–Bablok regression analysis was performed to compare APTT‐SLA and APTT‐n (*n* = 8324). As shown in Figure 1A, the results showed a regression equation with a positive slope (y = 1.82x−17.40) and a Spearman's rank correlation coefficient rS = 0.859 (*p* < 0.001). Furthermore, Bland–Altman analysis showed a fixed bias of 8.84 (95% CI: 8.65–9.02) seconds between APTT‐SLA and APTT‐n, and a proportional bias (*r* = 0.263, *p* < 0.01) (Figure 1B).

### 3.2 Analysis of the Difference in the Measured Values of the Two APTT Reagents and Their Fluctuation factors

To clarify the factors related to the difference between the two APTT reagents, univariate analysis was first performed using ΔAPTT as the objective variable and the other test items requested along with APTT. As shown in Table 2, the results showed a significant difference in 28 items. Next, multiple regression analysis was performed by test items with a significant difference and the correlation coefficient of ΔAPTT > |0.2|. As a result, albumin–globulin ratio (AGR), CRP, hematocrit (Hct), and PT were determined as independent factors (Table 3). In the analysis, matrix correlation was performed in advance to avoid multicollinearity. In Hct, red blood cell count (RBC), and hemoglobin concentration (Hgb), where the explanatory variables were highly correlated, Hct alone was retained. Additionally, while leaving PT for prothrombin time (PT), PT%, and PT‐INR and AGR for total protein (TP), ALB, and AGR, others were excluded from the explanatory variables.

**TABLE 2:** Basic statistics of test items and results of univariate analysis between test items and ΔAPTT

	n	Median (IQR)	rS	*p*‐Value
CRP (μg/L)	7161	7000 (37,000)	0.665	< 0.001
PT (s)	7845	12.7 (2.2)	0.324	< 0.001
PT‐INR	7845	1.1 (0.2)	0.311	< 0.001
WBC (10^9^/L)	7738	6.70 (4.30)	0.201	< 0.001
γ ‐GT (IU/L)	6459	34.0 (57.0)	0.172	< 0.001
CRE (μmol/L)	7538	68.1(38.0)	0.154	< 0.001
UN (mmol/L)	7520	5.36 (3.93)	0.119	< 0.001
LD (IU/L)	7200	223 (98.5)	0.094	< 0.001
ALP (IU/L)	6824	242 (148)	0.09	< 0.001
AST (U/L)	7542	24.0 (20.0)	0.066	< 0.001
MCV (fL)	7738	91.3 (7.20)	0.047	< 0.001
T‐BIL (μmol/L)	6671	10.2 (6.84)	0.029	0.019
ALT (IU/L)	7544	18.0 (22.0)	0.028	0.013
MCH (pg)	7738	30.6 (2.6)	0.011	<0.001
MCHC (g/L)	7738	336 (14)	−0.05	<0.001
K (mmol/L)	7515	4.0 (0.6)	−0.057	<0.001
PLT (10^9^/L)	7738	213 (116)	−0.067	<0.001
eGFR	7167	69.6 (37.6)	−0.114	<0.001
Cl (mmol/L)	7515	105 (4.0)	−0.129	<0.001
CK (IU/L)	6249	70.0 (87.0)	−0.138	<0.001
Na (mmol/L)	7515	140 (4.0)	−0.153	<0.001
TP (g/L)	7145	68 (13)	−0.23	<0.001
Hgb (g/L)	7738	118 (34)	−0.284	<0.001
RBC (10^12^/L)	7738	3.87 (1.11)	−0.286	<0.001
Hct (/L)	7738	0.35 (0.1)	−0.292	<0.001
PT% (%)	7845	88 (32)	−0.325	<0.001
ALB (g/L)	7222	34 (12)	−0.486	<0.001
AGR	7035	1.05 (0.50)	−0.53	<0.001

Abbreviations: AGR, albumin–globulin ratio; ALB, albumin; ALP, alkaline phosphatase; ALT, alanine aminotransferase; AST, aspartate aminotransferase; CRE, creatinine; CRP, C‐reactive protein; eGFR, estimated glomerular filtration rate; creatine kinase; Hct, hematocrit; Hgb, hemoglobin; IQR, interquartile range; LD, lactate dehydrogenase; MCH, mean corpuscular hemoglobin; MCHC, mean corpuscular hemoglobin concentration; MCV, mean corpuscular volume; PLT, platelet; PT, prothrombin time; PT‐INR, Prothrombin Time‐International Normalized Ratio; RBC, red blood cell; rS, Spearman's rank correlation coefficient; T‐Bil, total bilirubin; TP, total protein; UN, urea nitrogen; WBC, white blood cell; γ‐GT, γ ‐glutamyl transpeptidase.

**TABLE 3:** Results for multiple regression analysis on factors associated with ΔAPTT (*n* = 6557)

Variables	Unstandardized Coefficientsβ	Standard Error (β)	Standardized Coefficients β	*p*‐Value	95.0% Confidence Interval	
					Lower	Upper
Constant	12.139	0.565			11.032	13.247
AGR	−5.025	0.274	−0.225	<0.001	−5.562	−4.488
CRP	0.510 × 10^−4^	0.016× 10^−4^	0.365	<0.001	0.479 × 10^−4^	0.542 × 10^−4^
Hct	−7.8	1.37	−0.065	<0.001	−10.49	−5.11
PT	0.220	0.017	0.132	<0.001	0.186	0.253
WBC	−0.001	0.010	−0.001	0.942	−0.021	0.019

*Note*: Adjusted coefficient of determination *R* ^2^ = 0.327.

Abbreviations: AGR, albumin–globulin ratio; CRP, C‐reactive protein; Hct, hematocrit; PT, prothrombin time; WBC, white blood cell.

### 3.3 Effects of CRP and ALB additions

**FIGURE 2:**
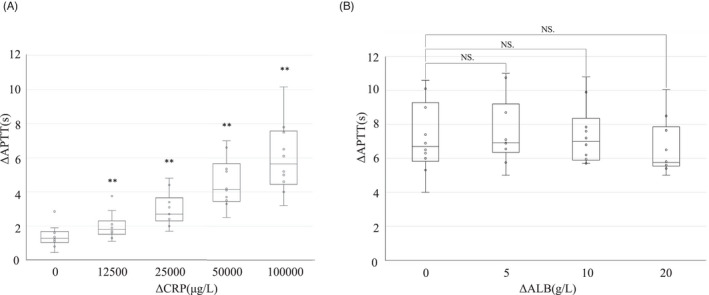
Effects of CRP and ALB addition to the APTT measured values. (A) Differences in APTT were measured after adding purified CRP to the pooled plasma to achieve ΔCRP of 12,500, 25,000, 50,000, and 100,000 μg/L. (B) Differences in APTT were measured after adding ALB to the pooled plasma to achieve ΔALB of 5, 10, and 20 g/L. no significant: Data are mean ± 95 confidence intervals (*n* = 10 for each group). **p < 0.01 versus 0 (control). ALB, Albumin; CRP, C‐reactive protein; NS, no significant; ΔALB, Change in ALB; ΔAPTT, Coagpia APTT‐n measured value ‐ Thrombocheck APTT‐SLA measured value; ΔCRP, Change in CRP.

In order to confirm the possibility of the direct effects of CRP and the AGR on the reaction system of APTT, which were associated with ΔAPTT in the multiple regression analysis, samples with different values of these CRP and ALB were prepared, and a spiking experiment was conducted. As a result, the addition of CRP significantly increased the ΔAPTT in the sample of ΔCRP 1.25 mg/dl, and ΔAPTT increased in a concentration‐dependent manner (Figure 2A). Whereas, despite the addition of ALB to change the AGR, no significant change was observed in ΔAPTT (Figure 2B).

### 3.4 Effects of phospholipid addition

**FIGURE 3:**
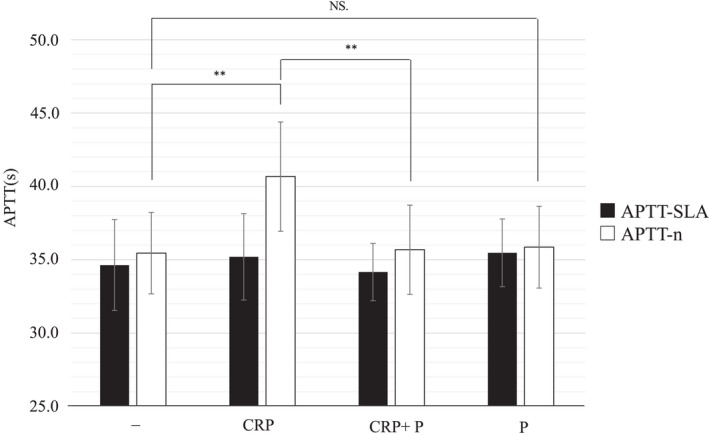
Effect of addition of phospholipid on the APTT measured value. APTT was measured using two reagents, APTT‐SLA and APTT‐n, after purified CRP (CRP), purified CRP + phospholipid (CRP + P), and phospholipid (P) were added to the pooled plasma. Data are mean ± 95.0% confidence intervals (*n* = 10 for each group). **p < 0.01. APTT‐n, Coagpia APTT‐n; APTT‐SLA, Thrombocheck APTT‐SLA; CRP, C‐reactive protein; NS, no significant.

Previous studies have reported that CRP inhibits phospholipids in some APTT reagents and causes a pseudo‐prolongation of APTT.^9^ Therefore, in order to investigate whether CRP inhibits phospholipids in the reagent, purified CRP was added to phospholipid added samples and the effects on APTT values were examined. As shown in Figure 3, sample with plasma and CRP showed a significant prolongation of APTT‐n as compared with to samples without the addition of CRP nor phospholipids. Instead, the addition of both CRP and phospholipids canceled the prolongation of APTT‐n. Furthermore, neither APTT‐SLA nor APTT‐n showed significant effects when phospholipids alone were added.

## 4 DISCUSSION

In this study, we analyzed the dissociation in the measured values discovered the parallel measurement of APTT‐n and APTT‐SLA, and by using clinical laboratory data in medical records, we have performed multiple regression analysis and revealed that the dissociation in the measured values between the two reagents was partly caused by CRP. In addition, from the spiking experiment of CRP, we have found out that the prolongation the measured values were prolonged in APTT‐n compared to APTT‐SLA. This suggested that one of the reasons for could be caused by the interaction of CRP on phospholipids in APTT‐n reagent.

APTT is most commonly measured in blood coagulation screening test, reflecting the actions of intrinsic coagulation factors and common coagulation factors. However, in the present day, in addition to detect these abnormalities, APTT measurement has significant meaning in the detection of lupus anticoagulant (LA) and monitoring test of unfractionated heparin, and its test purposes have become complicated and diversified. Along with the diversification, APTT reagents with various susceptibility characteristics have been developed.^10^ However, due to the diversification of the composition and origin of contact factor activators and phospholipids, each APTT reagent has different sensitivities to coagulation factors, and it has been reported that the selection of reagents has a major impact on measurement results.^4^, ^5^, ^6^, ^7^, ^11^, ^12^ Therefore, as we previously performed a parallel measurement of APTT‐SLA and APTT‐n and observed some dissociated samples, we focused on the analysis of other test items requested at the same time as APTT by performing multiple regression analysis to clarify the factors related to the difference in the measured values of the two APTT reagents.

In multiple regression analysis, a positive correlation with CRP, negative correlation with the AGR, and prolongation of PT were detected as factors related to the difference in measured values of the two APTT reagents. Since Hct affects the coagulation factor activity according to the ratio of anticoagulant to plasma CLSI guideline (H21‐A5) suggests to adjust the liquid volume of sodium citrate in samples with hematocrit exceeding 55%.^13^ PT is also commonly measured in blood coagulation screening test, detecting abnormalities of extrinsic coagulation factors, and reflects an activity of coagulation factors in a sample.^14^ Therefore, it is presumed that Hct and PT were extracted as related factors, because of the difference in coagulation factor sensitivity of the reagents.

Whereas, it was uncertain whether CRP and AGR directly affect the APTT measurements. Therefore, we next prepared samples with different values for these two items and conducted a spiking experiment to examine the direct effects on the APTT reaction system regarding the AGR, since ALB is the main component of plasma protein, and ALB alone showed a negative correlation with ΔAPTT nearly equal to the AGR, ALB was added to change the AGR for the examination. CRP addition showed a significant increase in ΔAPTT, prolonging the measured values of APTT‐n in a concentration‐dependent manner. CRP is one of the acute reactive proteins and is widely used in daily clinical practice as a marker of inflammation. However, it has been reported that for some APTT reagents, CRP can be a direct cause of a pseudo‐prolongation of APTT.^9^ On the other hand, although the AGR showed a negative correlation with ΔAPTT in multiple regression analysis, no significant change was observed even when AGR was changed by ALB addition. This suggests that the AGR does not directly affect the APTT reaction system. One of the factors that lowers the AGR is the decrease in ALB, and it has been reported that factors related to coagulation such as fibrinogen, factor V, and factor VIII fluctuate in hypoalbuminemia.^15^, ^16^ Therefore, it was considered that the AGR was detected as a related factor of ΔAPTT because it possibly reflected the difference in the sensitivity to such coagulation factors fluctuations.

Finally, previous studies have reported that CRP binds to some phospholipids.^9^, ^17^ Furthermore, since the phospholipids composed in APTT‐SLA and APTT‐n vary; synthetic phospholipids, and phospholipids derived from a rabbit brain, we have focused on phospholipids, and addition of phospholipids was examined. Results showed that the prolongation caused by CRP was canceled by the addition of phospholipid, and it was presumed that the binding of CRP to the phospholipid in the APTT‐n reagent triggered the prolongation of APTT. To summarize, suggested that ΔAPTT was caused by the difference of the phospholipid component and its interaction with CRP.

Our study has certain limitations. First, test items of each coagulation factor activity, which has been reported to be a factor in the difference in the measured values of the two APTT reagents, could not be used as a variable in the multiple regression analysis because the number of test requests was significantly lower than for APTT. Second, the prolongation of APTT‐n by CRP was canceled by the addition of phospholipid, suggesting that the phospholipids in the reagents had an effect on APTT‐n. However, because the composition of phospholipids in the reagents was heterogeneous, we could not examine the mechanism in detail.

## 5 CONCLUSION

As purposes to measure APTT have diversified, new APTT reagents, that has the property of being able to sensitively capture a specific pathological condition, have been developed. However, in conditions with coagulation factors fluctuate in a complex manner, unexpected difference in measured values between reagents may be observed. From this study, it was considered that the dissociation in the measured values from the result of the parallel measurement of the two reagents was partly caused by the interaction of CRP with the phospholipids in the APTT‐n reagent, leading to a pseudo‐prolongation of APTT. APTT is often requested for testing in inflammatory conditions, and when using reagents such as APTT‐n, it should be noted that it can be pseudo‐prolonged due to CRP. In addition, it is important to understand the characteristics of reagents used at each own facility. We hope that the results of this study will contribute to standardize the APTT reagent.

## Data Availability

The data that support the findings of this study are available from the corresponding author upon reasonable request.
